# Urban–Rural Differences in Patterns and Associated Factors of Multimorbidity Among Older Adults in China: A Cross-Sectional Study Based on Apriori Algorithm and Multinomial Logistic Regression

**DOI:** 10.3389/fpubh.2021.707062

**Published:** 2021-08-30

**Authors:** Chichen Zhang, Shujuan Xiao, Lei Shi, Yaqing Xue, Xiao Zheng, Fang Dong, Jiachi Zhang, Benli Xue, Huang Lin, Ping Ouyang

**Affiliations:** ^1^School of Health Management, Southern Medical University, Guangzhou, China; ^2^Department of Health Management, Nanfang Hospital, Southern Medical University, Guangzhou, China; ^3^Institute of Health Management, Southern Medical University, Guangzhou, China

**Keywords:** multimorbidity, urban–rural, older adults, health management, associated factors, patterns

## Abstract

**Introduction:** Multimorbidity has become one of the key issues in the public health sector. This study aimed to explore the urban–rural differences in patterns and associated factors of multimorbidity in China and to provide scientific reference for the development of health management strategies to reduce health inequality between urban and rural areas.

**Methods:** A cross-sectional study, which used a multi-stage random sampling method, was conducted effectively among 3,250 participants in the Shanxi province of China. The chi-square test was used to compare the prevalence of chronic diseases among older adults with different demographic characteristics. The Apriori algorithm and multinomial logistic regression were used to explore the patterns and associated factors of multimorbidity among older adults, respectively.

**Results:** The findings showed that 30.3% of older adults reported multimorbidity, with significantly higher proportions in rural areas. Among urban older adults, 10 binary chronic disease combinations with strong association strength were obtained. In addition, 11 binary chronic disease combinations and three ternary chronic disease combinations with strong association strength were obtained among rural older adults. In rural and urban areas, there is a large gap in patterns and factors associated with multimorbidity.

**Conclusions:** Multimorbidity was prevalent among older adults, which patterns mainly consisted of two or three chronic diseases. The patterns and associated factors of multimorbidity varied from urban to rural regions. Expanding the study of urban–rural differences in multimorbidity will help the country formulate more reasonable public health policies to maximize the benefits of medical services for all.

## Introduction

Aging has now become a global trend, a trend that is likely to continue in the future (1). China has become one of the most rapidly aging countries in the world (2). According to data from the National Bureau of Statistics of China, there were 253.88 million older adults aged 60 years and above in China at the end of 2019, accounting for 18.1% of the total population (3). With the accelerated population aging, more attention must be paid to the health issues of older adults. Multimorbidity is regarded as one of the key issues in the global public health sector and has also become a prominent health problem among Chinese older adults (4). Multimorbidity is defined as two or more chronic diseases in one person at a certain time (5). Compared with a single chronic disease, the treatment difficulty, medical consumption, economic burden, and risk of death among older adults with multimorbidity were increased (6–9). Multimorbidity is also associated with lower functional status (10), poor quality of life, and well-being (11, 12). The health management and clinical treatment of older adults with multimorbidity have, therefore, become major challenges (13).

Since the foundation of the People's Republic of China in 1949, China has enforced a stringent urban and rural household registration system. The reasons for the formation of the urban–rural dichotomy are historical continuity, as well as institutional changes and national development strategies. With the adoption of the reform and opening up policy in 1978, the urban and rural household registration system of China was progressively dismantled, but the urban–rural dual track system still has a significant influence on the economy and society of China (14). There are significant differences in income, social resource allocation, and access to welfare policies between urban and rural areas of China due to differences in their urban and rural economic development levels, household registration system design, and employment types (the rural population is primarily engaged in agricultural activities, while the urban population is primarily engaged in industry and service) (15). There are many family tragedies in the vast rural areas of China, particularly, in the economically underdeveloped regions, where older adults commit suicide, indicating the poor situation of older adults in rural areas (16). In 2018, the per capita disposable income of urban residents in China was CNY¥39,251, and that of rural residents was CNY¥14,617, which is a large gap (17). In addition, another study has documented the trends and gaps in health disparities between urban and rural areas (18). From a nationwide perspective, there may be significant urban–rural differences in patterns and associated factors of multimorbidity among older adults in China.

Previous studies focused predominantly on the prevalence, health care utilization, and health-associated outcomes of multimorbidity among older adults (19–22). As findings from a study done in China suggest, multimorbidity is more prevalent among the rural population (58.3%) than among their urban counterparts (50.4%) (23). Despite advances, rural China has less access to quality healthcare than urban China (18), as the majority of rural primary care doctors still lack a full university degree (24). Moreover, the patterns of multimorbidity have also gradually developed into a hot topic of current research. Noe et al. study (25) described patterns of multimorbidity in low-, middle-, and high-income countries, which were limited to 12 chronic diseases. Furthermore, another study also indicated that chronic diseases often occur in pairs among older adults in China, especially those with hypertension or dyslipidemia (26). However, there is limited evidence of differences in patterns of multimorbidity between urban and rural areas in China. In terms of the economy, social security, health services, and infrastructure, there are huge gaps in urban–rural areas of China (27), so it is necessary to explore the patterns of multimorbidity among older adults in urban and rural areas of China.

A previous study showed that the burden of multimorbidity is mainly caused by a series of associated factors (28). Well-established factors associated with multimorbidity are age, gender, body mass index (BMI), and education level (29, 30). However, the previous studies on the associated factors of multimorbidity among older adults mostly focused on the individual-level factors. In order to comprehensively understand the factors associated with multimorbidity, we cannot just focus on individual-level characteristics and have to consider other contextual factors, such as the environment, society, and policy level based on the health ecological model (31). Given the fact that the economic development of Chinese cities is significantly faster than that of rural areas (32), it is particularly important to study the different risk factors for multimorbidity among older adults in urban and rural areas.

An increasing number of studies have been undertaken to explore the patterns and associated factors of multimorbidity in China. However, there is limited evidence of differences in patterns and associated factors of multimorbidity between urban and rural areas, which may not be conducive to improving health management. In a period of rapid social transformation, the development is extremely uneven between urban and rural areas (18). It can be expected that the patterns and associated factors of multimorbidity may vary between urban and rural older adults. There is a dearth of evidence on urban–rural differences in patterns and associated factors of multimorbidity in China, which is a very important consideration in determining comprehensive intervention strategies applicable to rural and urban areas (33).

Given the significant disparities in the resource allocation and welfare systems between urban and rural areas in China, these disparities may be closely related to multimorbidity prevention and control. In such a context, this study aimed to identify the urban–rural differences in patterns and associated factors of multimorbidity among older adults and to provide a scientific reference for comprehensive intervention strategies and health management measures applicable to both rural and urban areas.

This study will be helpful in promoting dynamic multimorbidity prevention and control. The research on the patterns and associated factors of multimorbidity in China, as the largest developing country in the world, could be useful in revealing the link between social resource allocation and multimorbidity, particularly, in developing or undeveloped areas of the world.

## Materials and Methods

### Sample and Participants

A questionnaire-based cross-sectional study was conducted in Shanxi province, which comprises a total of 11 cities (Taiyuan, Datong, Yangquan, Changzhi, Jincheng, Shuozhou, Jinzhong, Yuncheng, Xinzhou, Linfen, and Lvliang) from June to August 2019. All the participants were interviewed face-to-face using a structured questionnaire by trained interviewers with medical knowledge. In order to get a representative sample of older adults, we used a multi-stage stratified cluster sampling method to select the participants in 11 cities. The sampling method was as follows: first, according to the order of districts (counties) on the website of the Shanxi province government, each district (county) in every city was numbered. Second, two districts (counties) in each city were selected using the random number table, and then two communities (administrative villages) were drawn from each district (county) in the same way. Last, considering that the different residential communities (natural villages) contain different numbers of older adults, only one residential community (natural village) was randomly selected from each community (administrative village), when the size of the number of older adults in the residential community (natural village) met the research requirements. If the number of older adults drawn from the residential community (natural village) is not enough, another residential community (natural village) is randomly selected as a sampling unit for supplementation. Finally, we randomly selected older adults who met the criteria in this study.

The inclusion criteria for this study were: (1) participants aged 60 years and above, and (2) having clear awareness and barrier-free communication skills. Those who had difficulty communicating were excluded. A total of 3,266 questionnaires were distributed, of which 3,250 respondents effectively completed the questionnaires; thus, the effective response rate was 99.51%.

All the study procedures were approved by the university ethics committee. All participants were informed of the purpose and procedure of the research upon their recruitment and assured of their right to refuse to participate. Their anonymity and confidentiality were guaranteed. After signing the consent, the participants were invited to participate in a face-to-face questionnaire where trained investigators collected the data.

### Instruments

The questionnaire consisted of two sections: self-made general information and types of chronic diseases. To illustrate the variety of factors that may be associated with multimorbidity among older adults, the health ecological model was adopted as the theoretical framework. Considering the difficulty of quantifying the macro-system policy environment indicators, this study only summarized the independent variables in four aspects, as follows: (1) personal characteristics: age, residence, family history of genetics, and BMI; (2) behavioral characteristics: regularity of three meals, frequency of fresh fruit consumption, smoking status, and drinking status, and sleep quality; (3) interpersonal network: marital status and empty nest status; (4) socio-economic status: educational level and monthly income. Theoretically, this analysis model better illustrates the factors associated with multimorbidity of older adults with different characteristics, but it is limited by the existing literature.

The questionnaire on chronic diseases includes 26 chronic diseases (e.g., hypertension, diabetes, rheumatoid, or rheumatoid arthritis). The information about chronic diseases was collected through self-reporting, which was based on the diagnostic evidence of medical records or the prescriptions from doctors.

### Statistical Analysis

The data were analyzed using IBM SPSS Version 24.0 Statistical software (IBM, NY, USA). The chi-square test was used to compare the prevalence of chronic diseases among older adults with different demographic characteristics. Confidence level with *P* < 0.05 was considered statistically significant. The Apriori algorithm in IBM SPSS Modeler Version 18.0 software was employed to analyze common patterns of multimorbidity among the older adults in the study, which mined valuable patterns in large, unordered data as association rules. It is essential to discover interesting and close correlations between items based on a large amount of data. As a data mining technology, association rules can help researchers extract valuable knowledge from huge data sets. Three kernel values are involved with association rule analysis, such as support, confidence, and lift (34). Based on this study, the support of A → B was the probability of the simultaneous occurrence of chronic diseases A and B. The confidence was the conditional probability of suffering from chronic disease B under the premise of suffering from chronic disease A. The degree of lift reflects the influence of the consequent B on the antecedent A compared to the overall. Therefore, when the degree of lift L_A−B_ > 1, A → B can be considered as a directional association. In the study, set the minimum conditional support to 3.0%, the minimum rule confidence to 30%, and the maximum number of preceding items to five. Multinomial logistic regression was used to examine the relationship between multimorbidity and potential associated factors. The level of significance was *P* < 0.05 (two-tailed test).

## Results

### Prevalence of Urban–Rural Differences of Chronic Diseases Among Older Adults

**Figure 1:**
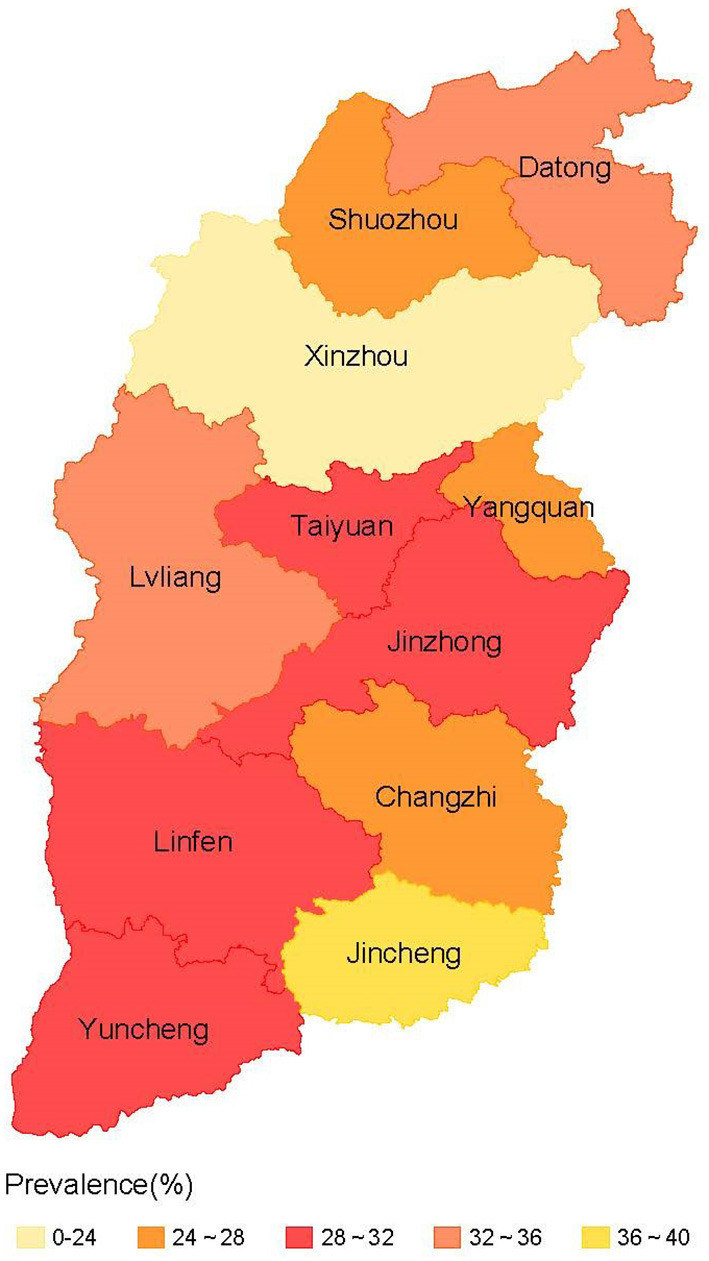
The prevalence of multimorbidity in 11 cities of Shanxi province.

Among the 3,250 older adults surveyed, 1,901 (58.5%) had chronic diseases and 985 (30.3%) had multimorbidity. The prevalence of multimorbidity among older adults in the 11 cities of Shanxi province is shown in Figure 1. Moreover, 26.7% of the respondents had multimorbidity in urban areas compared with 33.2% in rural areas. The number of multimorbidity ranged from 2 to 9. The coexistence of 2, 3, and 4 chronic diseases was relatively common, accounting for 54.1, 26.2, and 11.2%, respectively. The 10 most prevalent chronic diseases were hypertension, diabetes, rheumatoid or rheumatoid arthritis, hearing impairment, digestive system diseases, osteoporosis, coronary heart disease, eye diseases, respiratory diseases (bronchitis, emphysema, asthma, etc.), and stroke. Table 1 shows the urban–rural differences and Table 2 shows the gender differences in the 10 most prevalent chronic diseases among older adults (1).

**Table 1:** The urban–rural differences of the 10 most prevalent chronic diseases among older adults.

**Chronic diseases**	**Urban (** ***n*** **=** **1,453)**		**Rural (** ***n*** **=** **1,797)**		***X*^**2**^**
	**N (%)**	**order**	**N (%)**	**order**	
Hypertension	426 (29.3)	1	531 (29.5)	1	0.021
Diabetes	179 (12.3)	2	173 (9.6)	3	6.029^*^
Rheumatoid or rheumatoid arthritis	114 (7.8)	3	220 (12.2)	2	16.844^***^
Coronary heart disease	95 (6.5)	4	109 (6.1)	8	0.305
Hearing impairment	92 (6.3)	5	171 (9.5)	4	10.952^**^
Digestive system diseases	79 (5.4)	6	132 (7.3)	6	4.820^*^
Osteoporosis	76 (5.2)	7	133 (7.4)	5	6.291^*^
Eye diseases	65 (4.5)	8	117 (6.5)	7	6.308^*^
Respiratory diseases	51 (3.5)	9	94 (5.2)	9	5.582^*^
Stroke	45 (3.1)	10	85 (4.7)	10	5.580^*^

* *P < 0.05*,

** *P < 0.01*,

*** *P < 0.001*.

**Table 2:** The gender differences of the 10 most prevalent chronic diseases among older adults.

**Chronic diseases**	**Male (** ***n*** **=** **1,515)**		**Female (** ***n*** **=** **1,735)**		***X*^**2**^**
	**N (%)**	**order**	**N (%)**	**order**	
Hypertension	422 (27.9)	1	535 (30.8)	1	3.459
Diabetes	159 (10.5)	2	193 (11.1)	3	0.331
Rheumatoid or rheumatoid arthritis	126 (8.3)	4	208 (12.0)	2	11.824^***^
Coronary heart disease	79 (5.2)	7	125 (7.2)	6	5.445^*^
Hearing impairment	133 (8.8)	3	130 (7.5)	5	1.799
Digestive system diseases	95 (6.3)	5	116 (6.7)	8	0.230
Osteoporosis	71 (4.7)	9	138 (8.0)	4	14.350^***^
Eye diseases	59 (3.9)	10	123 (7.1)	7	15.617^***^
Respiratory diseases	81 (5.3)	6	64 (3.7)	9	5.215^*^
Stroke	77 (5.1)	8	53 (3.1)	10	8.660^**^

* *P < 0.05*,

** *P < 0.01*,

*** *P < 0.001*.

### Urban–Rural Differences in Association Rules for Multimorbidity

The results of the Apriori algorithm showed that 10 combinations of binary chronic diseases with strong association strength were obtained among older adults in urban areas. Eleven combinations of binary chronic diseases and three combinations of ternary chronic diseases with strong association strength were obtained among older adults in rural areas (Table 3). The patterns of multimorbidity were dominated by hypertension, atherosclerosis, and other chronic diseases among older people in both urban and rural areas. In addition, seven multimorbidity patterns associated with rheumatic or rheumatoid arthritis were found in rural older adults.

**Table 3:** Analysis results of urban–rural differences in association rules for multimorbidity.

**Consequent**	**Antecedent**	**Support (%)**	**Confidence (%)**	**Lift**
**Urban**				
Hypertension	Atherosclerosis	3.44	60.00	2.05
Hypertension	Stroke	3.10	55.56	1.90
Hypertension	Coronary heart disease	6.54	52.63	1.80
Hypertension	Diabetes	12.32	52.51	1.79
Hypertension	Eye diseases	4.47	50.77	1.73
Hypertension	Osteoporosis	5.23	47.37	1.62
Hypertension	Rheumatic or rheumatoid arthritis	7.85	39.47	1.35
Hypertension	Hearing Impairment	6.33	36.96	1.26
Coronary heart disease	Atherosclerosis	3.44	34.00	5.20
Diabetes	Atherosclerosis	3.44	34.00	2.76
**Rural**				
Hypertension	Coronary heart disease	6.07	55.05	1.86
Hypertension	Diabetes	9.63	53.18	1.80
Hypertension	Stroke	4.73	45.88	1.55
Hypertension	Eye diseases	6.51	43.59	1.48
Hypertension	Hearing Impairment	9.52	40.35	1.37
Hypertension	Hearing Impairment, Rheumatic or rheumatoid arthritis	3.28	38.98	1.32
Rheumatic or rheumatoid arthritis	Osteoporosis	7.40	36.84	3.01
Hypertension	Osteoporosis	7.40	35.34	1.20
Rheumatic or rheumatoid arthritis	Hearing Impairment	9.52	34.50	2.82
Rheumatic or rheumatoid arthritis	Hearing Impairment, Hypertension	3.84	33.33	2.72
Rheumatic or rheumatoid arthritis	Respiratory diseases	5.23	32.98	2.69
Hearing Impairment	Rheumatic or rheumatoid arthritis, Hypertension	3.90	32.86	3.45
Hypertension	Rheumatic or rheumatoid arthritis	12.24	31.82	1.08
Hypertension	Digestive system diseases	7.35	30.30	1.03

### Univariate Analysis of Associated Factors Underlying Multimorbidity

The chi-square test showed that there were significant differences in age, residence, family history of genetics, education level, monthly income, marital status, empty nest status, frequency of fresh fruit consumption, smoking status, drinking status, BMI, sleep quality, and anxiety of multimorbidity. However, there was no statistically significant difference in the regularity of three meals of multimorbidity among older adults (Table 4).

**Table 4:** Comparison of the types of chronic diseases among older adults.

**Characteristics**	**Types of chronic diseases [** ***N*** **(%)]**			***X^**2**^***	***P***
	**None**	**1**	**≥2**		
**Personal characteristics:**					
**Age**				127.342	< 0.001
60~	872 (64.6)	468 (51.1)	429 (43.6)		
70~	386 (28.7)	372 (40.6)	406 (41.2)		
80~	91 (6.7)	76 (8.3)	150 (15.2)		
**Residence**				18.231	<0.001
Urban	651 (48.3)	414 (45.2)	388 (39.4)		
Rural	698 (51.7)	502 (54.8)	597 (60.6)		
**Family history of genetics**				88.183	<0.001
Yes	15 (1.1)	56 (6.1)	95 (9.6)		
No	1,334 (98.9)	860 (93.9)	890 (90.4)		
**BMI**				30.269	<0.001
Underweight	204 (15.1)	116 (12.7)	145 (14.7)		
Normal	780 (57.8)	460 (50.2)	499 (50.7)		
Overweight	365 (27.1)	340 (37.1)	341 (34.6)		
**Behavioral characteristics:**					
**Regularity of three meals**				1.292	0.524
Yes	1,207 (89.5)	829 (90.5)	876 (88.9)		
No	142 (10.5)	87 (9.5)	109 (11.1)		
**Frequency of fresh fruit consumption**				28.986	<0.001
Eat almost every day	544 (40.3)	315 (34.4)	292 (29.6)		
Other	805 (59.7)	58 (6.3)	80 (8.1)		
**Smoking status**				60.782	<0.001
Smoking	281 (20.8)	177 (19.3)	171 (17.4)		
Quit smoking	81 (6.0)	138 (15.1)	138 (14.0)		
Never	987 (73.2)	601 (65.6)	676 (68.6)		
**Drinking status**				49.057	<0.001
Drinking	262 (19.4)	164 (17.9)	156 (15.8)		
Quit drinking	62 (4.6)	102 (11.1)	115 (11.7)		
Never	1,025 (76.0)	650 (71.0)	714 (72.5)		
**Sleep quality**				102.456	<0.001
Poor	177 (13.1)	201 (21.9)	298 (30.3)		
Good	1,172 (86.9)	715 (78.1)	687 (69.7)		
**Anxiety**				121.837	<0.001
Yes	383 (28.4)	271 (29.6)	482 (48.9)		
No	966 (71.6)	645 (70.4)	503 (51.1)		
**Interpersonal Network:**					
**Marital status**				11.459	<0.01
Married	1,061 (78.7)	705 (77.0)	716 (72.7)		
Other	288 (21.3)	211 (23.0)	269 (27.3)		
**Empty nest status**				42.610	<0.001
No children	51 (3.8)	9 (1.0)	10 (1.0)		
Empty nest	595 (44.1)	483 (52.7)	503 (51.1)		
Non-empty nest	703 (52.1)	424 (46.3)	472 (47.9)		
**Socio-economic status:**					
**Educational level**				62.654	<0.001
Elementary education and below	587 (43.5)	491 (53.6)	561 (57.0)		
Secondary education	596 (44.2)	364 (39.7)	364 (37.0)		
Higher education and above	166 (12.3)	61 (6.7)	60 (6.1)		
**Monthly income**				17.390	0.002
Low (<1,000 RMB)	654 (48.5)	476 (52.0)	561 (57.0)		
Middle (1,000~5,000 RMB)	632 (46.8)	393 (42.9)	386 (39.1)		
High (>5,000 RMB)	63 (4.7)	47 (5.1)	38 (3.9)		

### Results of Multinomial Logistic Regression

The results from multinomial logistic regression are shown in Table 5. Older age was identified as a significant risk factor for a single chronic disease (urban *OR*: 1.47, 95% *CI*: 1.10–1.97; rural *OR*: 1.57, 95% *CI*: 1.21–2.03) and multimorbidity (urban *OR*: 2.07, 95% *CI*: 1.52–2.80; rural *OR*: 1.64, 95% *CI*: 1.26–2.12). Family history of genetics was the most important independent predictor for a single chronic disease (urban *OR*: 6.53, 95% *CI*: 2.79, 15.28) and multimorbidity (urban *OR*: 13.53, 95% *CI*: 5.91, 30.98; rural *OR*: 7.98, 95% *CI*: 3.61–17.66). Those participants with elementary education and below had higher odds of multimorbidity compared with higher education and above graduates (urban *OR*: 1.72, 95% *CI*: 1.10–2.69; rural *OR*: 2.55, 95% *CI*: 1.20–5.41). Marital status had no significant impact on a single chronic disease and multimorbidity, regardless of urban–rural status. No child was found as a protective factor against multimorbidity in urban areas, but in rural areas, empty nesting was a risk factor for multimorbidity. Frequency of fresh fruit consumption had no significant impact on multimorbidity in rural areas, but in urban areas, another frequency was associated with higher odds of multimorbidity compared with eating almost every day (*OR*: 1.37, 95% *CI*: 1.04–18.2). Quit smoking and quit drinking were the associated factors of a single chronic disease and multimorbidity regardless of urban–rural status. As for BMI, being overweight was a significant predictor for a single chronic disease (*OR*: 1.84, 95% *CI*: 1.38–2.44) and multimorbidity (urban *OR*: 1.69, 95% CI: 1.25–2.30) in urban areas. Sleep quality was not significantly associated with a single chronic disease and multimorbidity in urban areas. But in rural areas, those with poor sleep had higher odds of a single chronic disease (*OR*: 2.11, 95% CI: 1.53–2.91) and multimorbidity (*OR*: 2.15, 95% CI: 1.59–2.92). The participants with anxiety symptoms were associated with higher odds of multimorbidity both in urban and rural areas (urban *OR*: 1.65, 95% *CI*: 1.22–2.23; rural *OR*: 2.09, 95% *CI*: 1.63–2.68).

**Table 5:** Multinomial logistic regression for having a single chronic disease and multimorbidity.

	**Urban**				**Rural**			
	**0 vs. 1**		**0 vs**. **≥2**		**0 vs. 1**		**0 vs**. **≥2**	
	**OR**	**95% CI**	**OR**	**95% CI**	**OR**	**95% CI**	**OR**	**95% CI**
**Personal characteristics**								
**Age (ref. 60** **~** **)**								
70~	1.47	(1.10–1.97)^*^	2.07	(1.52–2.80)^***^	1.57	(1.21–2.03)^**^	1.64	(1.26–2.12)^***^
80~	1.15	(0.70–1.90)	2.64	(1.65–4.24)	1.36	(0.83–2.21)	2.60	(1.69–4.00)
**Family history of genetics (ref. No)**								
Yes	6.53	(2.79–15.28)^***^	13.53	(5.91–30.98)^***^	5.47	(2.40–12.50)	7.98	(3.61–17.66)^***^
**BMI (ref. Normal)**								
Underweight	0.99	(0.65–1.53)	1.40	(0.92–2.13)	0.96	(0.68–1.37)	0.89	(0.64–1.26)
Overweight	1.84	(1.38–2.44)^***^	1.69	(1.25–2.30)^**^	1.43	(1.09–1.88)	1.23	(0.95–1.61)
**Behavioral characteristics**								
**Frequency of fresh fruit consumption (ref. Eat almost every day)**								
Other	1.14	(0.87–1.48)	1.37	(1.04–1.82)^*^	1.39	(1.05–1.84)^*^	1.31	(0.99–1.72)
**Smoking status (ref. Never)**								
Smoking	1.07	(0.72–1.58)	1.03	(0.68–1.54)	1.02	(0.71–1.44)	0.81	(0.57–1.15)
Quit smoking	2.42	(1.53–3.82)^***^	1.87	(1.15–3.02)^*^	2.43	(1.52–3.90)^***^	2.06	(1.29–3.29)^**^
**Drinking status (ref. Never)**								
Drinking	1.07	(0.72–1.57)	1.39	(0.93–2.07)	0.90	(0.62–1.31)	0.80	(0.55–1.17)
Quit drinking	1.94	(1.17–3.19)^*^	2.22	(1.33–3.71)^**^	2.89	(1.67–5.00)^***^	2.98	(1.74–5.09)^***^
**Sleep quality (ref. Good)**								
Poor	1.27	(0.88–1.82)	1.69	(1.19–2.40)	2.11	(1.53–2.91)^***^	2.15	(1.59–2.92)^***^
**Anxiety (ref. No)**								
Yes	0.99	(0.73–1.34)	1.65	(1.22–2.23)^**^	0.85	(0.65–1.11)	2.09	(1.63–2.68)^***^
**Marital status (ref. Other)**								
Married	0.65	(0.46–0.91)	0.99	(0.68–1.43)	1.24	(0.92–1.55)	0.94	(0.71–1.24)
**Empty nest status (ref. Non–empty nest)**								
No children	0.26	(0.09–0.79)^*^	0.30	(0.10–0.96)^*^	0.42	(0.14–1.23)	0.38	(0.14–1.07)
Empty nest	1.49	(1.15–1.94)^**^	1.20	(0.91–1.59)	1.20	(0.94–1.53)	1.29	(1.01–1.64)^*^
**Working and living environment**								
**Educational level (ref. Higher education and above)**								
Elementary education and below	1.40	(0.92–2.14)	1.72	(1.10–2.69)^*^	5.33	(2.09–13.59)^***^	2.55	(1.20–5.41)^*^
Secondary education	1.31	(0.89–1.92)	1.27	(0.84–1.94)	3.21	(1.25–8.24)^*^	1.97	(0.92–4.20)

* *P < 0.05*,

** *P < 0.01*,

*** *P < 0.001*.

## Discussion

The current study provides new evidence to determine the urban–rural disparity in patterns and associated factors of multimorbidity among older adults in China. The results of the current study indicated that the prevalence of multimorbidity among rural older adults (33.2%) was higher than the urban older adults (26.7%) in the study, which is significantly lower than the previous study (rural: 58.3%; urban: 50.4%) in China (23). The inconsistency may be partly due to the differences in sampling methods, sampling size, and type of chronic disease information. A comparative analysis between rural and urban areas shows that the rural areas are at a disadvantage in the prevalence of multimorbidity. Compared with urban older adults, rural older adults have lower socio-economic status, poorer social services, and lower access to quality medical services, all of which may contribute greatly to their high prevalence of multimorbidity.

The study also pointed out that under the influence of the urban–rural dual structure, older adults living in rural areas had more complex patterns of multimorbidity compared with older adults living in urban areas based on the Apriori algorithm. There were more association rules pointing to osteoporosis in the rural older population than in the urban older population, and rheumatoid and rheumatoid arthritis were present in the antecedents of several association rules pointing to osteoporosis, which is in line with the results of a previous study (35). It may be largely explained by the fact that most rural older adults suffer from malnutrition as well as the need to do heavy farm work. On the one hand, attention should be paid to the bone and joint health of rural older adults. On the other hand, among patients with osteoporosis, extra attention should be paid to the comorbidity of rheumatoid arthritis for better screening and prevention.

Regardless of urban or rural areas, older age, family genetic history, lower education level, quitting smoking, quitting drinking, and anxiety are all associated factors for multimorbidity among older adults. With the increase in age, the immune function of older adults gradually weakens, and the longer the body is exposed to various associated factors, the higher the risk of multimorbidity. Family genetic history is the most significant risk factor for multimorbidity. Some studies have reported an association between family genetic history and the risk of chronic disease (36, 37). A previous study established a strong relationship between anxiety and multimorbidity (38), which was in line with the previous study showing a significant association between anxiety and multimorbidity (39).

However, there is still some variation in the factors associated with multimorbidity between urban and rural areas. In urban areas, the unique protective factor of multimorbidity is childlessness, and the unique risk factor is insufficient fresh fruit intake and high BMI. It is worth noting that being childless may prevent urban older adults to suffer from multimorbidity. They do not have to spend a lot of money and energy on cultivating their children. Hence, they have more freedom to do their own things and participate in more social activities, thereby keeping good physical health. Compared with rural areas, urban residents have been shown to exhibit higher rates of unhealthy diet, physical inactivity, and obesity (40, 41). The rapid improvement in the living conditions of urban residents is the primary factor leading to their being overweight. Moreover, the modernization of the urban industrial structure has led to a sharp decline in the proportion of manual labor, which has led to an increase in the incidence of obesity. Reasonable dietary behavior and physical exercise can enhance physical fitness and reduce the occurrence of being overweight.

In rural areas, the unique risk factors are empty nest status and poor sleep quality. In rural China, the traditional concept of “bringing up their children for old age” of older adults is relatively strong (42). Bringing up their children for old age refers to the hard work of parents pulling their children to grow up. When they are old, grown-up children can understand the rewards and take care of the older parents without leaving them alone and helpless. With the development of urbanization, an increasing number of rural young people migrate into urban areas, while their parents are left in rural areas, which may make their parents feel lonely and have less daily care (27). Older adults are at more risk of multimorbidity if they are under empty nest status. Additionally, since most rural residents are engaged in high-intensity agricultural activities every day, it is not conducive to their health. Especially during the busy agricultural season, residents get up early and stay up late, and sleep quality is not guaranteed, which may be the main reason for their increased risk of multimorbidity. Therefore, older adults should try to get enough sleep to prevent the occurrence of chronic diseases. Overall, effective prevention and control measures should be developed from multiple perspectives and at multiple levels, in order to reduce the prevalence of multimorbidity and improve the health of older adults in both urban and rural areas.

### Policy Implications

The current study findings have important implications for public health policy and planning. Due to the development imbalance caused by the urban–rural dichotomy, older adults in rural areas have much less access to healthcare than their urban counterparts. Expanding the study of urban–rural differences in multimorbidity will help the country formulate more reasonable public health policies to maximize the benefits of medical services for all. First, since the rural population has a higher prevalence of multimorbidity, the priority for health system transformation to address multimorbidity lies in the rural areas, where the needs are greatest and service providers are less skilled and more sparsely distributed. Second, it is necessary to strengthen the shared construction of regional public service facilities to achieve an effective interface between urban and rural areas. Third, it is necessary to build an integrated urban–rural medical insurance system to achieve regional equality at the national level. Moreover, it is recommended to implement relevant preferential policies to encourage and guide medical school graduates to work in rural areas, which can improve the capacity of primary care services in rural areas. Finally, considering the importance of primary care in addressing multimorbidity in a coordinated and continuous manner, it seems critical to strengthen primary care so that equally good-quality integrated services can be provided to both rural and urban people of China.

### Limitations

The current study had various strengths and limitations. The strengths of the study are the large sample size and regions. Additionally, a comparative analysis between rural and urban areas suggests that rural areas are at a disadvantage in multimorbidity, which distinguishes itself from the previous studies. Although this study had certain value for the prevention and control of multimorbidity in older adults, it also had limitations. First, due to the cross-sectional design, there were information deviations when investigating and analyzing behavioral lifestyles. In addition, the prevalence of chronic diseases was self-reported, and thus, there may be information bias. To clarify these findings, longitudinal studies are necessary. Finally, the data were collected from older adults in Shanxi province, China, and other populations need to be verified in future studies.

## Conclusion

Multimorbidity is more prevalent among rural older adults than urban older adults in the study. Future health system development in China should transform from preventing and controlling a single chronic disease to addressing the multimorbidity of older adults. The priority for such a transformation is rural areas. Moreover, there are some differences in the patterns and factors associated with multimorbidity in urban and rural older adults. Specifically, empty nest status, frequency of fresh fruit consumption, BMI, and sleep quality, have different impacts on multimorbidity in urban and rural areas. Therefore, intervention measures for multimorbidity among older adults in urban and rural areas should be differentiated. The current study findings may have important implications for the intervention programs aimed at narrowing the gap in multimorbidity between urban and rural older adults. The allocation of resources needs changes to maintain a balance between rural and urban regions. First, it is necessary to strengthen the shared construction of regional public service facilities to achieve an effective interface between urban and rural areas. Second, it is necessary to integrate urban and rural welfare systems (medical insurance, disability care, and medical assistance) to achieve regional equality at the national level.

## Data Availability Statement

The original contributions presented in the study are included in the article/Supplementary Material, further inquiries can be directed to the corresponding author/s.

## Ethics Statement

The studies involving human participants were reviewed and approved by the Shanxi Medical university ethics committee. All participants were informed of the purpose and procedure of the research upon their recruitment, and assured of their right to refuse to participate. Their anonymity and confidentiality were guaranteed. After signing the consent, participants were invited to conduct questionnaires face to face to collect data by trained investigators. The patients/participants provided their written informed consent to participate in this study.

## Author Contributions

CZ and SX conceived the idea. FD, YX, and BX participated in data collection and statistical analysis. SX drafted the manuscript. HL, JZ, and PO edited the paper. LS and XZ gave many valuable comments on the draft and polished it. All authors have read and approved the manuscript.

## Conflict of Interest

The authors declare that the research was conducted in the absence of any commercial or financial relationships that could be construed as a potential conflict of interest.

## Publisher's Note

All claims expressed in this article are solely those of the authors and do not necessarily represent those of their affiliated organizations, or those of the publisher, the editors and the reviewers. Any product that may be evaluated in this article, or claim that may be made by its manufacturer, is not guaranteed or endorsed by the publisher.
